# Pregabalin for the Treatment of Neuropathic Pain: A Systematic Review of Patient-Reported Outcomes

**DOI:** 10.7759/cureus.70443

**Published:** 2024-09-29

**Authors:** Zhihui Wang, Iffat Naeem, Tinu Oyenola, Ahmad Raza Khan, Amanda Dennis, Samuel Obamiyi, Emilie Toews, Shilpa Singh, Gebin Zhu

**Affiliations:** 1 Research, Health Research Partners, Calgary, CAN; 2 Orofacial Pain, University of Rochester School of Medicine and Dentistry, Rochester, USA; 3 Dental Medicine, Texas Tech University Health Sciences Center El Paso, El Paso, USA; 4 Dentistry, St. John’s Community Health, Los Angeles, USA

**Keywords:** neuropathic pain, patient-reported outcomes, pregabalin, quality of life, sleep disturbance

## Abstract

Neuropathic pain (NeP) arises from pathologies of the nervous system, significantly impacting patient functionality and quality of life. Pregabalin is a first-line treatment for NeP, but there has been limited focus on patient-reported outcomes (PROs). This systematic review synthesizes PROs from clinical studies assessing pregabalin's efficacy in treating NeP, with a focus on sleep disturbance, mental health, and health-related quality of life (HRQOL). Following Cochrane Collaboration guidelines, we conducted a systematic search of randomized controlled trials and epidemiological studies reporting PROs associated with pregabalin treatment for NeP. Sixteen studies met the inclusion criteria and were narratively synthesized. The findings indicate that pregabalin significantly improved sleep interference and HRQOL across multiple studies, particularly at doses of 300 mg/day or higher. However, improvements in mental health outcomes, such as anxiety and depression, were inconsistent across studies. No meta-analysis was conducted due to the heterogeneity of outcomes. In conclusion, while pregabalin shows robust efficacy in reducing NeP-related sleep disturbances, its effects on mental health and HRQOL are less consistent. These findings highlight the need for a more holistic approach to NeP treatment, incorporating both clinical outcomes and PROs.

## Introduction and background

Neuropathic pain (NeP) is a disorder of the nervous system resulting from altered mechanisms operating in the peripheral nervous system, spinal cord, and supraspinal levels and defined by the International Association for the Study of Pain as pain arising from the pathologies of the nervous system, which causes alterations in the function, chemistry, and structure of neurons [1]. NeP affects 2-8% of the world population and can have a significant influence on the patient’s functional abilities and quality of life. NeP can result from spinal cord injury, tumors of the brain and spinal cord, and other diseases affecting the nervous system [2]. NeP can be a secondary condition to extremely prevalent conditions such as diabetes, stroke, cancer, herpes zoster virus infection, and autoimmune diseases [3].

Pregabalin has become a leading neuromodulation agent to treat NeP after its approval by both the European Union and the FDA (US Food and Drug Administration) in 2004. Pregabalin has shown promising results in both clinical and preclinical studies in managing NeP. Treatment outcomes have shown pregabalin to be a reliable anti-analgesic, is easy to use, and has high tolerability in patients, with reports of an improved safety profile as compared to opioids and tricyclic antidepressants [4]. Clinical and preclinical studies have mapped the various treatment outcomes associated with pregabalin, which show largely positive results in control and of central and peripheral NeP. Treatment is dose-dependent, and data suggests no need for higher doses of pregabalin to control long-term pain [5,6].

Recent reviews and meta-analyses have largely focused on pain outcomes in the treatment of NeP with pregabalin [7]. However, little focus has been given to patient-reported outcomes (PROs) associated with the clinical use of pregabalin, which can include wellness, physical health, sleep, mental health, and health-related quality of life (HRQOL). With the growing importance of patient-centered healthcare systems, PROs can be a valuable source of information on patient well-being that cannot otherwise be discerned using technology or healthcare providers [1]. PROs cover a broad set of measures that include direct subjective assessments by the patient about their health, including symptoms, function, well-being, HRQOL, and perception and satisfaction with treatment [8]. Prioritizing and exploring the subjective PROs of pregabalin use has the potential to provide a more holistic picture of treatment effects considering the patients' experience with treatment and the influences of patient intrinsic values and expectations, along with the overall effectiveness of pregabalin as NeP treatment as interpreted by the patient [8]. Further, a more comprehensive understanding of pregabalin treatment and its associated benefits and harms can inform clinical practice, including the need for supportive care and comparison to other therapies to treat the disorder [9]. In addition, the relationship between NeP and a decline in HRQOL measures and other measures of patient functioning, therefore, requires consideration when formulating treatment plans with pregabalin [10].

A preliminary search of PROSPERO, MEDLINE, Cochrane Database of Systematic Reviews, and JBI Evidence Synthesis was conducted, and no current or underway scoping reviews or systematic reviews on synthesizing PROs in the treatment of NeP were discovered. This review aims to fill this gap by conducting a systematic synthesis to explore the various PROs reported by patients in the treatment of NeP outside of measures of pain. A systematic review provides a comprehensive synthesis of existing research, minimizing bias and offering high-level evidence by aggregating and critically evaluating findings from multiple studies on a specific topic [11]. The objectives of this systematic review are twofold: to summarize the various PROs reported by patients receiving pregabalin treatment for NeP and discuss the treatment effects of pregabalin on the above PROs as reported by study participants.

## Review

Methods

This systematic review was conducted in accordance with criteria outlined by The Cochrane Handbook for Systematic Reviews, which provides stepwise guidance on conducting high-quality rapid reviews [12]. The criteria laid out by this group informed the research question development, setting and eligibility criteria, study search, study selection, data extraction, risk of bias assessment, and synthesis of the study results. Additionally, in order to increase the transparency of the results, this review followed the Preferred Reporting Items for Systematic Review and Meta-Analyses (PRISMA) [13]. This review will feature a systematic synthesis of the included studies without a meta-analysis of the results.

Search Strategy

The search strategy aimed to locate published primary studies in major databases, including MEDLINE, Embase, CINHAL, Cochrane Database of Systematic Reviews, and CENTRAL. An initial limited search of MEDLINE (PubMed) was undertaken to identify studies on the topic (see Appendices). The text words contained in the titles and abstracts of relevant articles and the index terms used to describe the articles were used to develop a full search strategy with an information specialist for search in the appropriate databases (see Appendices for the MEDLINE search strategy). The search strategy, including all identified keywords and index terms, was translated for each included information source. The reference lists of pertinent review articles selected for full-text review were screened for additional papers. Additional limitations on the search strategy included published literature only in humans, in English, and those published between the years 2000 and 2024. Records published from gray literature sources will not be included.

Inclusion Criteria

This review followed a preachified inclusion and exclusion criteria, outlined in Table 1. For the population, this review will consider studies that include adult participants 18 years of age or older, excluding studies focusing on children or adolescents, animals, or cell cultures. Participants will be included from all settings. The study population must have defined diagnostic criteria for NeP and will include those who suffer from NeP as informed by the International Association for the Study of Pain. Therefore, this can include patients suffering from multiple disorders, with NeP being an outcome (e.g., diabetic neuropathy, HIV-related neuropathy). This review considered studies that explore treatment with pregabalin of NeP. Studies were included if they assessed the treatment of NeP with pregabalin with a placebo comparator or comparators consisting of other therapies for NeP. Only studies assessing treatment with pregabalin monotherapy will be included.

**Table 1:** Inclusion and exclusion criteria for systematic review NeP: neuropathic pain, PRO: patient-reported outcomes, RCTs: randomized controlled trials

	Included	Excluded
Population	Humans	In vitro studies
	Adult participants as defined by the inclusion criteria of the study being reviewed	Animal studies
	All patient populations suffering from central or peripheral NeP	Studies on children and adolescents
		Studies in patients with fibromyalgia, sciatica, chemotherapy-related NeP, preoperative patients, or neuropathy with no pain outcomes
Intervention	Treatment of NeP with pregabalin (pregabalin monotherapy)	Combined therapy of NeP with pregabalin and other types of treatment
Comparator	Placebo	Comparator not described
	Other standard therapies	Comparators with pregabalin included
Outcomes	PROs as reported by patients include HROL, mental health, mood, physical health, wellness, and psychological health	Pain associated outcomes
		Cost analysis outcomes
		PROs reported on adverse outcomes of treatment
Study design	Phase III, double-blind, placebo-controlled RCTs (efficacy studies)	Phase IV trials
	Any study size and duration of intervention	Cross-over RCTs
	Epidemiological studies with comparator group	Unblinded trials
		Modelling studies
		Commentaries
		Letters to editor
		Reviews
		Cross-sectional studies
		Observational studies
		Any study with no comparator group
Other restrictions	Country: no restrictions	
	Date of publication: 2015-2024	

The primary outcome of interest was PROs that measure various aspects of health and wellness as perceived by the patient, with no interpretation from the clinicians or researchers conducting the study. Outcomes included physical health, psychological health, quality of life, sleep quality, and mood. It was expected that PROs would be measured using standardized criteria or questionnaires. In order to focus the review on PROs, pain-related outcomes were not considered in the systematic synthesis of this review.

This review considered entirely quantitative study designs, primarily randomized controlled trials (RCTs) within phase II or III clinical testing. Trials with any study size and duration were included in the analysis. If the RCT employed a cross-over design, it was not included in the analysis due to the risk of a carryover effect and high selection bias. Phase IV trials, which tend to be unblinded, were included in the analysis. The authors hypothesized that epidemiological studies (e.g., case-cohort studies, retrospective cohort studies) that utilize treatment and comparator groups will be rare but will be included in the analysis nonetheless. Any epidemiological studies that did not utilize a comparator group (e.g., cross-sectional, observational) were not included. Finally, systematic reviews were not included in the analysis of this review but were utilized for citation mining of relevant primary studies.

Study/Source of Evidence Selection

Following the search, all identified records were collated and uploaded into EndNote (Clarivate Analytics, PA, USA) screening software, and duplicates were removed. Following a pilot test, titles and abstracts of the de-duplicated record list were screened by two independent reviewers for assessment against the inclusion criteria for the review (Table 1). After the pilot test, all records were screened in the title/abstract stage. The full text of the included records was obtained and prepared for screening. After pilot testing screening, all full texts were screened by two reviewers for inclusion. Reasons for the exclusion of full-text papers that do not meet the inclusion criteria were recorded and reported in the review. Any disagreements that arose between the reviewers at each stage of the screening process were resolved through discussion or with a third reviewer. The results of the search were reported via the PRISMA flow diagram [14].

Data Extraction

Data were extracted from all full-text records that met the inclusion criteria to be included in the review by a single extractor using a prespecified data extraction tool developed in Excel (Microsoft Corporation, Redmond, WA, USA). The data extracted included aspects of study characteristics, which included study methods, duration of study, setting, population, details about the study population, pregabalin intervention details, and type of primary efficacy measures associated with pregabalin treatment. Additionally, all reported PROs from the included studies were extracted, along with the treatment effects of pregabalin in all treatment groups assessed.

Data Analysis and Presentation

Results were presented using a tabular format to list the type and definition of PROs utilized in the reviewed studies, along with the treatment effects of pregabalin treatment. Result tables were stratified with the patient etiology of NeP. Major results of the study in regard to PROs will be reported in both a tabular format and systematically to describe how the results relate to the review objective and questions.

Assessment of Methodological Quality

Eligible studies will be critically appraised by two independent reviewers at the study level using the Cochrane Collaboration tool for assessing the risk of bias in randomized trials (Higgins 2011). Any disagreements that arose between the reviewers were resolved through discussion or with a third reviewer. The results of the critical appraisal were reported in a table with an accompanying narrative. All studies, regardless of the results of their methodological quality, underwent data extraction and synthesis (where possible).

Results

Search Results

**Figure 1:**
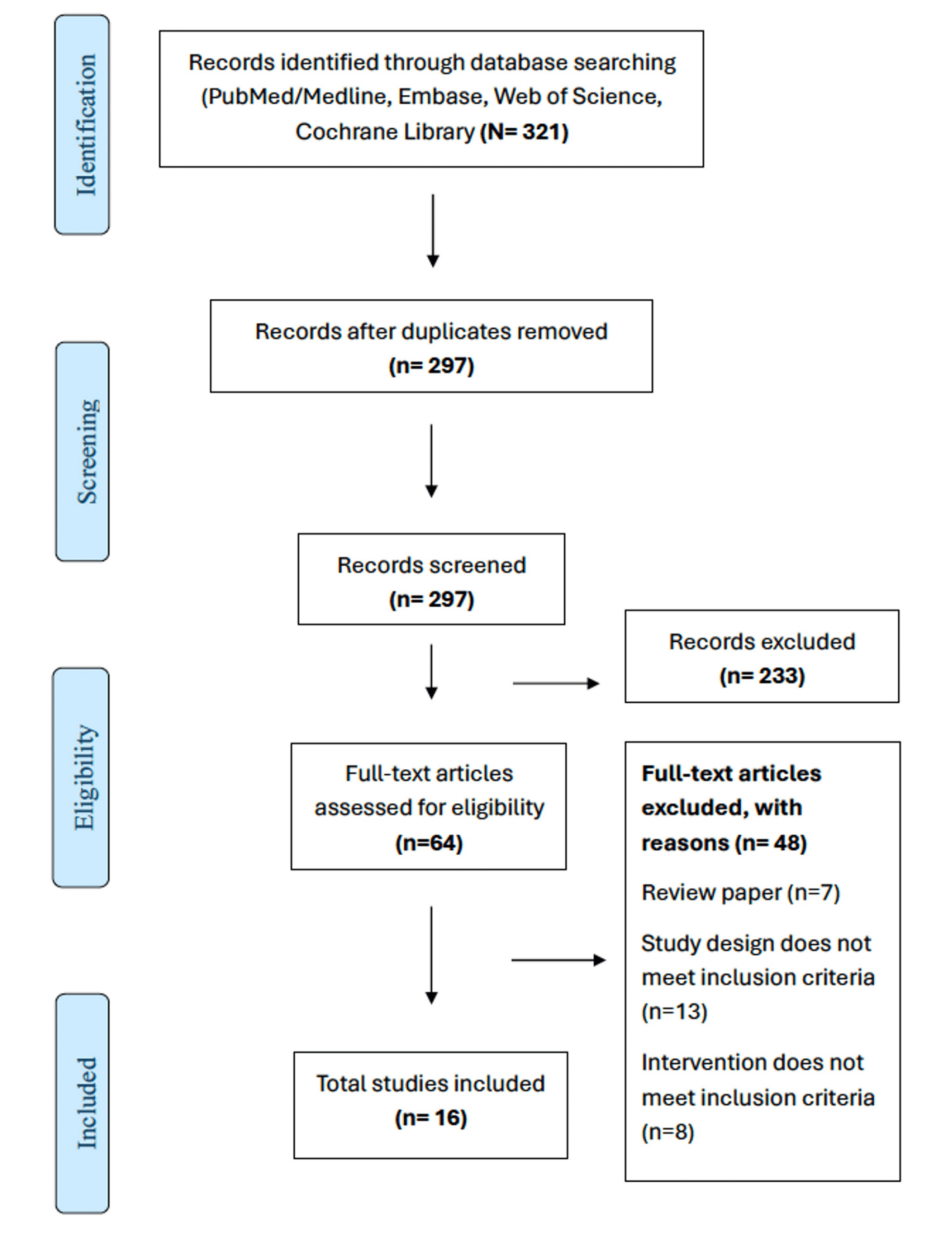
PRISMA flow diagram depicting the number of records screened at both the title/abstract stage and full-text stage. Reasons for exclusion at the full-text stage are also given PRISMA: Preferred Reporting Items for Systematic Review and Meta-Analyses

A total of 321 studies were screened in the title and abstract phases, and 297 were full-text screened. A final 16 studies were reviewed, and their reported PROs were systematically summarized (Figure 1) [15-30]. The summary of the study characteristics is reported in Table 1. Studies reporting PROs associated with pregabalin varied in their etiology of NeP, including studies with patient populations with diabetic peripheral neuropathy (DPN), postherpetic neuralgia (PHN), and NeP as a result of surgical or non-surgical traumatic events. All studies included participants over the age of 18, with randomized trials ranging from a study period of 4-20 weeks. All studies were placebo-controlled, except for two studies: one compared pregabalin with venlafaxine and carbamazepine, and another compared pregabalin with duloxetine and gabapentin. The treatment effects of pregabalin on the reports for all studies are reported in Table 2. Studies utilized various tools, surveys, and questionnaires to measure the PROs of pregabalin treatment for NeP, which have been summarized in Table 3.

**Table 2:** Study characteristics* * Not including patient-reported outcomes, see Table 3. SD: standard deviation, NeP: neuropathic pain, DPN: diabetic peripheral neuropathy, SF-MPQ: short-form McGill pain questionnaire, VAS: visual analog scale, NRS: numerical pain rating scale from patient diaries, CGIC: clinical global impression scale, PGIC: patient global impression scale, NR: not reported, PHN: postherpetic neuralgia, NPS: neuropathic pain scale, BPI: brief pain inventory short form, GESM: global evaluation of study medication, VAS-PI: visual analog scale pain intensity, PTNP: post-traumatic neuropathic pain, PDI: pain disability index, LTR: loss of therapeutic response, AE: adverse event, ECG: electrocardiogram

Study	Country	Study design and duration	Patient inclusion criteria	Pregabalin treatment (mg/day) and comparator	Sample size (N)	Age (mean (SD))	Male (%)	Baseline pain scores (mean (SD))	Efficacy measures
Patients with NeP as a result of DPN									
Arezzo et al. 2008 [15]	USA	Randomized, double-blind, placebo-controlled, parallel-group trial, 13 weeks	Patient with type 1 or 2 diabetes mellitus with DPN for more than 3 months and a score of >40 mm on SF-MPQ VAS	600	82	58.2 (9.6)	70.7	6.28 (1.47)	Daily pain diaries using 11-point NRS (0=no pain, 10=worst possible pain), SF-MPQ, CGIC, and PGIC
				Placebo	85	58.3 (10.9)	52.9	6.58 (1.58)	
Freynhagen et al. 2005 [16]	60 centres in nine European countries	Randomized, double-blind, multicenter, placebo-controlled, parallel-group trial, 12 weeks	Patients with a diagnosis of painful DPN due to type 1 or 2 diabetes mellitus OR PHN present for more than 3 months due to herpes zoster virus	Flexible dose 150-600	141	62.7 (10.6)	52.5	7.0 (1.5)	Daily pain diaries using 11-point NRS (0=no pain, 10=worst possible pain), PGIC
				Fixed dosed 600	132	61.8 (11.0)	54.5	7.1 (1.7)	
				Placebo	65	61.8 (11.0)	56.9	6.7 (1.7)	
Rosenstock et al. 2004 [17]	USA	Randomized, double-blind, multicenter, parallel-group comparison trial, 8-weeks	Patients with type 1 or type 2 diabetes reporting symmetrical painful symptoms in distal extremities (DPN) for 1-5 years. Score of >40 mm on SF-MPQ VAS. Average daily pain score of >4 on 11-point NRS	300	76	59.2 (12.3)	55.3	NR	Daily pain diaries using 11-point NRS (0=no pain, 10=worst possible pain), SF-MPQ, CGIC, and PGIC
				Placebo	70	60.3 (10.3)	57.1	NR	
Jiang et al. 2011 [18]	USA	Randomized, double-blind, placebo-controlled, 4 weeks	Diagnosis of DPN including distal symmetrical sensory loss of bilateral lower extremities. Moderate to severe average daily pain on VAS	Flexible dose 75-300	20	55.1 (14.36)	55	75.37^3^ (12.90)	Respiratory rate, posture, activity level, QRS complexes, and R-R intervals via a 3-axis accelerometer and a 3-lead, single-channel ECG, NPS, modified BPI
				Placebo	20	59.65 (21.5)	70	70.78 (18.76)	
Lesser et al. 2004 [19]	USA	Randomized, double-blind, placebo-controlled, parallel-group, multicenter study, 5 weeks	Diagnosis of type 1 or type 2 diabetes mellitus and distal symmetric sensorimotor polyneuropathy for 1 to 5 years. Score of >40 mm on SF-MPQ VAS	75	77	61.3 (10.5)	55.8	6.7 (1.3)	Daily pain diaries using 11-point NRS (0=no pain, 10=worst possible pain), CGIC and PGIC
				300	81	59.0 (9.2)	59.3	6.2 (1.4)	
				600	82	62.0 (9.7)	63.4	6.2 (1.5)	
				Placebo	97	57.8 (11.6)	60.8	6.6 (1.5)	
Raskin et al. 2014 [20]	USA	Phase 3B, multicenter, double-blind, placebo-controlled randomized withdrawal trial, 20 weeks	Diagnosis of painful DPN for more than 3 months due to type 1 and type diabetes mellitus with inadequate pain control. Score of >40 mm on SF-MPQ VAS and a score of >4 on 11-point NRS	300	147	58.8 (9.2)	51	6.8 (1.2)	Daily pain diaries using 11-point NRS (0=no pain, 10=worst possible pain), PGIC, BPI, and GESM
				Placebo	147	58.3 (10.5)	54.4	6.7 (1.3)	
Razazian et al. 2014 [21]	Iran	Randomized, double-blind, parallel-group, placebo-controlled, 4 weeks	Diagnosis of painful DPN for more than 3 months due to type 1 and type diabetes mellitus. Score of >40 mm on SF-MPQ VAS	75	86	55.4 (11.1)	34.7	NR	Subjective pain as assessed by the VAS-PI rated daily by patients, BPI
				Venlafaxine	86	55.1 (9.6)	36	NR	
				Carbamazepine	85	58.3 (10.4)	48.2	NR	
Satoh 2011 [22]	Japan	Randomized, placebo-controlled, 12 weeks	Diagnosis of painful DPN for 1 year due to type 1 and type diabetes mellitus with inadequate pain control. Score of >40 mm on SF-MPQ VAS and a score of >4 on 11-point NRS	300	134	61.3 (10.3)	56.7	6.0 (1.4)	Daily pain diaries using 11-point NRS (0=no pain, 10=worst possible pain), SF-MPQ, CGIC, and PGIC
				600	45	62.2 (10.3)	61.5	6.1 (1.3)	
				Placebo	135	61.3 (9.6)	60.1	6.1 (1.4)	
Tanenberg et al. 2011 [23]	43 study centers in the United States, Germany, Canada, and Puerto Rico	Phase 4, open-label, randomized, placebo-controlled, 12 weeks	Outpatients with type 1 or type 2 diabetes mellitus with DPN confirmed by a score of at least 3 on section B of the Michigan neuropathy screening instrument. Score of >4 on 11-point NRS	300	134	61.9 (10.7)	56.7	5.7 (1.4)	Daily pain diaries using 11-point NRS (0=no pain, 10=worst possible pain), BPI, PGIC
				Duloxetine + gabapentin	135	61.9 (10.8)	61.5	5.8 (1.5)	
				Duloxetine	138	60.9 (10.2)	60.1	5.8 (1.5)	
Tolle et al. 2008 [24]	58 centers in Europe (Germany, Hungary, Poland, and the United Kingdom), Australia, and South Africa	Randomized, double-blind, placebo-controlled, multicenter study, 12 weeks	Diagnosis of painful DPN for 1 year due to type 1 and type diabetes mellitus with inadequate pain control. Score of >40 mm on SF-MPQ VAS and a score of >4 on 11-point NRS	150	99	58.51 (12.4)	54.5	NR	Daily pain diaries using 11-point NRS (0=no pain, 10=worst possible pain), CGIC and PGIC
				300	99	57.28 (10.5)	53.5	NR	
				300/600	101	59.7 (11.3)	60.4	NR	
				Placebo	96	58.93 (11.7)	53.1	NR	
Patients with NeP as a result of PHN									
Huffman et al. 2017 [25]	USA	Randomized, double-blind, placebo-controlled, 13 weeks	Diagnosis of PHN (pain present for >3 months after herpes zoster infection. Average daily pain score of >4 on NRS	Flexible dose 82.5-660	208	61.9 (13.5)	35.6	6.8 (1.5)	Time to LTR, defined as <30% pain response relative to single-blind baseline or discontinuation due to AE or lack of efficacy, daily pain diaries using 11-point NRS (0=no pain, 10=worst possible pain), BPI
				Placebo	205	62.2 (13.9)	40.5	6.9 (1.4)	
Sabatowski et al. 2004 [26]	53 sites in Europe and Australia	Randomized, double-blind, placebo-controlled, 7 weeks	Patients with pain present for > 6 months after healing of the herpes zoster infection. Score of >40 mm on SF-MPQ VAS and a score of >4 on 11-point NRS	150	81	71.3 (10.1)	48	6.9 (1.7)	Daily pain diaries using 11-point NRS (0=no pain, 10=worst possible pain), SF-MPQ, CGIC, and PGIC
				300	76	71.9 (10.3)	41	7.0 (1.6)	
				Placebo	81	73.2 (10.3)	46	6.6 (1.6)	
Liu 2017 [27]	China	Randomized, double-blind, placebo-controlled, Phase III trial, 8 weeks	Diagnosed with PHN. Score of >40 mm on SF-MPQ VAS	300	111	65.7 (8.6)	51.4	NR	Daily pain diaries using 11-point NRS (0=no pain, 10=worst possible pain), SF-MPQ, CGIC, and PGIC
				Placebo	109	64.1 (9.6)	56.9	NR	
Patients with NeP as a result of surgical or non-surgical traumatic event									
Cardenas et al. 2013 [28]	Chile, China, Columbia, the Czech Republic, Hong Kong, India, Japan, the Philippines, the Russian Federation, and the United States	Randomized, double-blind, placebo-controlled, 12 weeks	Patients with C2-T12 spinal cord injury (SCI) of >12 months duration. Patients with SCI due to trauma, diving, ischemia, or surgery to remove benign tumors were included	Flexible dose 150-600	111	46.1 (12.7)	75.7	6.5 (1.45)	Duration-adjusted average change of daily pain diaries using 11-point NRS (0=no pain, 10=worst possible pain), PGIC
				Placebo	108	45.6 (13.8)	85.2	6.5 (1.41)	
Markman et al. 2018 [29]	USA	Randomized, double-blind, placebo-controlled, 15 weeks	PTNP for ≥ 6 months after a surgical or non-surgical traumatic event (e.g., history of a motor vehicle accident, fall, sports injury, knee or hip replacement, hernia repair, thoracotomy, mastectomy, focal/localized burns, or crush injury). Peripheral nerve(s) implicated in the pain were identified to confirm nerve trauma, and pain was categorized as neuropathic based on prespecified criteria. Score of >4 on 11-point NRS	Flexible dose 150-600	275	52.8 (12.9)	NR	6.41 (1.3)	Daily pain diaries using 11-point NRS (0=no pain, 10=worst possible pain), PGIC
				Placebo	267	53.5 (12.6)	NR	6.5 (1.3)	
Patients with NeP of unspecified etiology									
Vranken et al. 2008 [30]	Netherlands	Randomized, double-blind, placebo-controlled, 4 weeks	Patients suffering from severe NeP due to lesions or dysfunction in the central nervous system. NeP was described by at least one of the following: burning pain, paroxysmal episodes of shooting pain, or pain on light touch. The pain had to persist continuously for at least 6 months. Score of >6 on 11-point NRS	600	17	54.2 (9.4)	NR	7.6 (0.8)	Daily pain diaries using 11-point NRS (0=no pain, 10=worst possible pain), PDI
				Placebo	16	54.7 (9.7)	NR	7.4 (1.1)	

**Table 3:** Treatment effects of pregabalin on PROs PRO: patient-reported outcomes, NeP: neuropathic pain, DPN: diabetic peripheral neuropathy, NRS: numerical rating scale, MOS-SS: medical outcome study scale – sleep subscale, NR: not reported, SF-36: short-form health survey, POMS: profile of mood states questionnaire, CI: confidence interval, STAI: state-trait anxiety inventory scale, BDI: Beck depression inventory scale, SDS: Sheehan disability scale, QOL-DN: quality of life questionnaire-diabetic neuropathy version, BPI: brief pain inventory short form, EQ-5D: EuroQol-5 dimension, HADS: hospital anxiety and depression scale, ITT: intent-to-treat, PDI: pain disability index, HRQOL: health-related quality of life

Study	Treatment and comparator	PRO reported	Statistic reported	Outcome	Significance	General conclusions
Patients with NeP as a result of DPN						
Arezzo et al. 2008 [15]	Pregabalin and placebo	Pain-associated sleep interference using NRS recorded in daily sleep diaries	Mean treatment difference of sleep interference (95% CI)	-1.08 (-1.75, -0.41)	p=0.0019	Pregabalin showed robust efficacy for the treatment of painful DPN and pain-associated sleep interference
Freynhagen et al. 2005 [16]	Fixed/flexible dose of pregabalin and placebo	Pain-associated sleep interference using NRS recorded in daily sleep diaries	NR	NR	p<0.001	Flexible- and fixed-dose pregabalin were significantly superior to placebo in improving sleep-interference scores and both sleep disturbance and overall sleep problem index scores on the MOS-SS
		Overall sleep problem index from MOS-SS	NR	NR	p<0.05	
Rosenstock et al. 2004 [17]	Pregabalin and placebo	Pain-associated sleep interference using NRS recorded in daily sleep diaries.	Mean treatment difference of sleep interference (95% CI )	-1.54 (-2.28, -0.80)	p=0.0001	Analysis of sleep interference scores between the treatment groups showed statistically significant differences favoring pregabalin over placebo at the endpoint. In the SF-36, higher mean scores (i.e. more favorable) were reported for the pregabalin group compared to the placebo group in all of the health domains except role-physical. Changes in mean POMS score for the pregabalin-treated patients were more favorable for all mood state scales compared to placebo
		SF-36 scored from 0 to 100, with higher scores indicating better HRQOL	Mean difference (95% CI), mental health, vitality	3.47 (-1.73, 8.66), 3.24 (-2.13, 8.61)	p=0.1893, p=0.2343	
		POMS- 65 items assessing mood, tension, and other psychological symptoms rated on a 0–4 scale (0=applies not at all, and 4=applies extremely)	Mean difference (95% CI)	9.95 (-18.53, -1.37)	p=0.0234	
Jiang et al. 2011 [18]	Pregabalin and placebo	Spielberger STAI for symptoms of anxiety. Reduction in values means better health	Mean (SD), treatment, placebo state anxiety, trait anxiety	1.2 (3.28), 0.57 (5.91); 3.67 (5.51), 1 (6.16)	p=0.334, p=0.041	Compared with the placebo group, the pregabalin group had a greater reduction in STAI and BDI scores
		BDI for symptoms of depression. Reduction in score means better health	Mean (SD), treatment, placebo	-1.20 (1.57), 1.21 (4.25)	p=0.075	
		SDS - global functioning score. To evaluate functional impairment in 3 interrelated domains: work/school, social, and family life. Lower score means better health	Mean (SD), treatment, placebo	-7.73 (7.70), -1.67 (4.64)	p=0.019	
Lesser et al. 2004 [19]	Pregabalin (75, 300, 600 mg/day) and placebo	Pain-associated sleep interference using NRS recorded in daily sleep diaries	Mean difference (95% CI), 75 mg/day, 300 mg/day, 600 mg/day	0.01 (-2.43, 2.44), -4.89 (-7.29, -2.48), -5.18 (-7.58, -2.79)	p=0.9966, p=0.0001, p=0.0001	Pregabalin at a dose of 300,g/day and 600mg/day had significantly improved sleep interference as compared to placebo. Both the 300 and 600 mg/day pregabalin; groups were better than the placebo in the social functioning domain of the SF-36; the 300 mg/day pregabalin group had better results than the placebo group on the tension-anxiety mood scale of the POMS
		SF-36 – social functioning domain. Increased score means improvement	NR 75 mg/day, 300 mg/day, 600 mg/day	NR	p<0.05, p<0.01	
		POMS- 65 items assessing mood, tension, and other psychological symptoms rated on a 0–4 scale (0=applies not at all, and 4=applies extremely)	NR, 75 mg/day, 300 mg/day, 600 mg/day	NR	p<0.05	
Raskin et al. 2014 [20]	Pregabalin and placebo	QOL-DN - total scores. Negative values indicate improvement	Mean (SD), treatment, placebo	-20 (21.4), -14.4 (20.4)	p<0.05	Pregabalin was associated with a statistically significant improvement over placebo for 2 domains of the QOL-DN (physical functioning/large fiber and symptoms) and the total QOL-DN score, but not for the other domains. Pregabalin was associated with a statistically significant improvement over the placebo at a double-blind endpoint for the MOS-SS sleep disturbance subscale and overall sleep problems
		Sleep disturbance from MOS-SS	Mean (SD), treatment, placebo	-28.3 (24.4), -20.0 (26.6)	p<0.05	
Razazian et al. 2014 [21]	Pregabalin and venlafaxine and cabamazepine	BPI - mean score of sleep interference	Mean difference (95% CI) from Day 1 to end of the study, pregabalin, venlafaxine, carbamazepine	NR	p<0.001, p<0.001, p<0.001	At the endpoint, mean scores of work, mood, and sleep interference were significantly decreased in all 3 groups. The reduction of mean sleep and work interference scores in the pregabalin group were significantly higher than the carbamazepine and venlafaxine groups at day 35. Pregabalin and Venlafaxine were superior to carbamazepine on the mean scores for pain-related mood interference at day 35 but there is no statistically significant difference between pregabalin and venlafaxine
		BPI - mean score of mood interference	Mean difference (95% CI) from Day 1 to end of the study, pregabalin, venlafaxine, carbamazepine	NR	p<0.001, p<0.001, p<0.001	
		BPI - mean score of work interference	Mean difference (95% CI) from Day 1 to end of the study, pregabalin, venlafaxine, carbamazepine	NR	p<0.0001, p<0.0001, p<0.0001	
Satoh et al., 2011 [22]	Pregabalin (300, 600 mg/day) and placebo	Pain-associated sleep interference using NRS recorded in daily sleep diaries	Mean sleep interference scores; 300 mg/day, 600 mg/day	NR	p<0.0001, p<0.0273	The mean sleep interference scores at the study endpoint were significantly improved in the 300 and 600 mg⁄ day groups compared with placebo. The 300 mg⁄ day group showed significant improvement on several MOS-SS subscales, including sleep disturbance quantity of sleep and overall Sleep Problems Index compared with the placebo group; the 600 mg/day group was significantly superior to placebo on sleep adequacy. Pregabalin 600 mg/day was significantly superior to the placebo group on social functioning and vitality in the SF-36
		MOS-SS	NR	NR		
		SF-36 to assess quality of life. Increased score means improvement. Social functioning vitality	NR	NR		
Tanenberg et al. 2011 [23]	Pregabalin, duloxetine + gabapentin and duloxetine	BDI-II	Mean (SD), pregabalin, duloxetine + gabapentin, duloxetine	-2.6 (0.5), -2.3 (0.5), -2.5 (0.5)	NR	Differences between treatments were not significant for the other BPI pain measures, clinical global Impression of severity, depressive symptoms, or the SDS global measure
		SDS	Mean (SD), pregabalin, duloxetine + gabapentin, duloxetine	-5.0 (0.7), -4.5 (0.7), -3.5 (0.7)	NR	
Tolle et al. 2008 [24]	Pregabalin (15, 300, 300/600) and placebo	Pain-associated sleep interference using NRS recorded in daily sleep diaries	Mean treatment difference (95% CI) 150 mg/day, 300 mg/day, 300/600 mg/day	-0.45 (-1.05, 0.15), -0.62 (-1.22, -0.02), -1.01 (-1.60, -0.41)	p=0.144, p=0.0844, p=0.003	Assessment of endpoint, pain-related sleep-interference scores indicated significant superiority of 600 mg/day pregabalin over placebo. At the endpoint, all three pregabalin dose groups had significantly better mean EQ-5D utility scores than the placebo group
		EQ-5D to assess quality of life. Higher scores indicate improved health status	Mean treatment difference (95% CI) 15 mg/day, 300 mg/day, 300/600 mg/day	0.10 (0.03, 0.16), 0.08 (0.01, 0.14), 0.14 (0.07, 0.20)	p=0.0092, p=0.0263, p=0.0003	
Patients with NeP as a result of PHN						
Huffman et al. 2017 [25]	Pregabalin and placebo	Pain-associated sleep interference using NRS recorded in daily sleep diaries	Mean (SD), treatment, placebo	-4.5 (2.4), -3.6 (2.4)	p<0.0001	Pregabalin CR significantly improved secondary outcome measures of weekly mean pain score (1-week recall), sleep interference, MOS-SS, and HADS, and these improvements were observed in both the single-blind and double-blind phases of the study. The benefit from treatment and satisfaction domains of the BSW scale were significantly more improved with pregabalin than with placebo but the trend to improvement on the willingness to continue subscale was not significant
		MOS-SS index I (sleep disturbance, sleep adequacy, respiratory impairment, somnolence)	Mean (SD), treatment, placebo	-19.6 (19.6), -17.2 (22.6)	p=0.0324	
		HADS	Mean (SD), treatment, placebo anxiety, depression	-2.0 (3.5), -1.2 (3.7), -2.1 (3.3 1.4 (3.6)	p=0.0154, p=0.0166	
		SF-36 to assess quality of life. Increased score means an improvement	Mean (SD), treatment, placebo physical component, mental component	7.7 (7.8), 6.3 (8.4); 6.6 (11.3), 5.9 (11.0)	p=0.0082, p=0.2907	
		Benefit, satisfaction, willingness to continue measure (BSW) (3 single-item measures to capture patients’ perception of treatment effect in terms of the relative benefit, their satisfaction, and willingness to continue therapy)	% of patients reporting positive response, treatment, placebo benefit, satisfaction, willingness to continue	99, 99, 96, 91, 88, 81	p=0.0161, p=0.0378, p=0.0901	
Sabatowski et al. 2004 [26]	Pregabalin (150, 300 mg/day) and placebo	Pain-associated sleep interference using NRS recorded in daily sleep diaries	Mean treatment difference (95% CI), 150 mg/day, 300 mg/day	-1.11 (-1.71, -0.51), -1.43 (-2.04, -0.82)	p=0.0003, p=0.0001	Pregabalin also significantly improved mean sleep interference scores among the ITT population. Each of the eight domains of the SF-36 was analyzed separately. The 150 and 300 mg/day pregabalin groups were significantly superior to placebo in the mental health domain. While the 150 mg/day pregabalin group showed numerically greater improvement than placebo on the Zung self-rating depression scale index, the 300 mg/day pregabalin group showed statistically significant improvement
		SF-36 to assess quality of life. Increased score means an improvement	Treatment, placebo 150 mg/day, 300 mg/day	NR	p=0.043, p=0.043	
		Zung self-rating depression scale	Treatment, placebo 300 mg/day	NR	p=0.024	
Liu et al. 2017 [27]	Pregabalin and placebo	Pain-associated sleep interference using NRS recorded in daily sleep diaries	Mean treatment difference (95% CI)	0.54 (0.20)	p=0.0079	Pregabalin improved the secondary outcome measures of pain, sleep interference, SF-MPQ VAS, and SF-MPQ PPI significantly more than placebo at the endpoint. While improvements in the subscales of snoring, awakening short of breath, optimal sleep, sleep adequacy, somnolence, and sleep problems index with pregabalin were not significantly greater than placebo, statistically significantly greater improvements with pregabalin were observed in the MOS-sleep subscales of sleep disturbance and quantity of sleep compared with placebo
		Overall sleep problem index from MOS-SS	Mean treatment difference (95% CI)	-2.84 (-6.63, 0.94)	p=0.1403	
Patients with NeP as a result of surgical or non-surgical traumatic event						
Cardenas et al. 2013 [28]	Pregabalin and placebo	Pain-associated sleep interference using NRS recorded in daily sleep diaries	Mean treatment difference (95% CI)	-1.08 (0.26)	p<0.001	Improvements over placebo for both pain and pain-related sleep interference scores were evident after 1 week of pregabalin treatment and were sustained throughout the trial. Treatment with pregabalin also resulted in improvement over placebo on sleep disturbance, awakening shortness of breath, sleep quantity, and optimal sleep subscales of the MOS-SS, as well as the overall sleep problems index. Improvements over placebo were also evident for the depression subscale of the HADS at endpoint
		Overall sleep problem index from MOS-SS	Mean treatment difference (95% CI)	-8.67 (2.99)	p=0.004	
		HADS – anxiety subscale	Mean treatment difference (SD)	0.68 (0.43)	p=0.116	
		HADS – depression subscale	Mean treatment difference (SD)	0.99 (0.45)	p=0.029	
		Optimal sleep in pregabalin patients versus placebo	OR (95% CI)	2.81 (1.44, 5.49)	p=0.002	
Markman et al. 2018 [29]	Pregabalin and placebo	Pain-associated sleep interference using NRS recorded in daily sleep diaries	Mean treatment difference (95% CI)	-0.43 (-0.71, -0.14)	p=0.0031	The weekly mean sleep interference score at week 15 showed significantly greater improvement in the pregabalin group than in the placebo group
Patients with NeP of unspecified etiology						
Vranken et al. 2008 [30]	Pregabalin and placebo	PDI – Interference of chronic pain with activities of daily living. Higher PDI scores indicate more perceived disability	Mean (SD), treatment, placebo	35.7 (14.9), 43.3 (14.7)	p=0.111	Follow-up observation showed no significant difference in PDI scores between the two groups. There were significant improvements in health status (EQ-5D). Pregabalin treatment led to a significant improvement in the bodily pain domain. In the other domains, higher scores (i.e., more favorable) were reported for the pregabalin group without reaching statistical significance
		EQ-5D to assess quality of life– Utility section. Higher scores indicate improved health status	Median (IQR), treatment, placebo	0.59 (0.52, 0.67), 0.06 (-0.17, 0.57)	p<0.001	
		SF-36 - social domain. Higher scores mean better health	Mean (SD), treatment, placebo social domain, emotional domain, mental domain	63.8 (20.2), 58.1 (23.7), 55.0 (43.6), 40.0 (46.6), 70.3 (18.8), 62.0 (21.3)	p=0.425, p=0.3, p=0.020	

Pregabalin and Sleep Disturbance

Sleep disturbance was the highest reported PRO within the studies reviewed (Table 2). Sleep disturbance was primarily measured by daily patient diaries using a numerical rating scale (NRS) from 0 to 11 (0=no pain, 11=worst possible pain). Pregabalin showed robust efficacy in reducing sleep interference scores as measured by the NRS in all studies. A study assessing lower doses of pregabalin (i.e., 75 mg/day) reported non-significant results in reducing pain-related sleep interference [19]. However, the efficacy of pregabalin in reducing pain-related sleep interference improved when a dose of pregabalin equal to or higher than 300 mg/day was utilized [19,22]. It should be noted, however, that a study did not show this improvement even at the 300 mg/day dosage [24]. When compared to other NeP medications in patients with DPN, pregabalin was not superior to venlafaxine and carbamazepine in reducing pain-related sleep interference [21].

Secondary measures of sleep disturbance included the medical outcome study scale - sleep subscale (MOS-SS) (see Table 3 for details). Overall sleep problems as measured by this scale were significantly reduced in pregabalin treatment groups as compared to placebo in patients with DPN [16,20,22] and NeP associated with surgical and non-surgical traumatic events [28]. However, a study in PHN patients showed improved scores on the MOS-SS; the improvements were not significant between the pregabalin treatment and placebo groups [25,27].

Mental Health, Anxiety, and Depression

Measures of mental health, anxiety, and depression were measured using a variety of instruments that have been verified and used for psychological diagnosis, screening, and symptom monitoring. A popular questionnaire used in DPN patients was the profile of mood states questionnaire (POMS), which found significantly positive influences of pregabalin for all mood states measured in the scale [17]. A study by Lesser et al. [19] found superior treatment effects for the POMS scale for 300 mg/day pregabalin groups as compared to the 75 and 600 mg/day groups. Assessment of psychological variables using POMS was not done in other patient groups within the reviewed studies (i.e., NeP due to PHN or traumatic events). Other measures of mood interference included a brief pain inventory of mood interference, which found significant improvement in the mean score of mood interference in all treatment groups assessed, with pregabalin and venlafaxine being superior to carbamazepine [21].

Measures for depressive symptoms included the Beck depression inventory (Table 2), which is a readily used 21-item inventory measuring the characteristics, attitudes, and symptoms of depression, where a reduction in inventory scores means better psychological health [31]. In patients with DPN, the treatment did not lead to a significant improvement in depressive symptoms compared to placebo, as assessed by the Beck depression inventory [18]. Conversely, in patients with PHN, a significant improvement was observed in the Zung self-rating depression scale index [32] for those receiving the treatment. Notably, one validated inventory used to measure affective psychological symptoms related to depression showed improvement with a dosage of 300 mg/day of pregabalin but not with 150 mg/day [26]. It should be noted, however, that a numerical improvement in inventory scores was noted for the 150 mg/day pregabalin study in the same study.

Spielberg state-trait anxiety inventory (STAI) [33] is an important tool utilized for assessing anxiety that helps to differentiate anxiety from depressive symptoms in clinical settings. A reduction in mean scores of the inventory indicates reduced anxiety. Studies involving DPN patients suggest pregabalin provides a greater reduction in anxiety compared to placebo, though significant differences were observed only for the trait anxiety (related to personality and individual differences) rather than the state anxiety (transient psychological and physiological reactions) component of the STAI [18]. For patients with PHN, the HADS [34] designed to assess anxiety and depression in the general medical population of patients with physical health issues has shown significant improvement both in anxiety and depression subscales for those receiving pregabalin [25]. However, a significant improvement in scores was not observed in patients with NeP resulting from traumatic events [28].

Quality of Life Measures

Multiple HRQOLs were utilized to understand the functioning of patients affected by NeP and the treatment effects of pregabalin. The 36-item short-form health survey (SF-36) was the most utilized survey in the studies reviewed. In DPN patients, some studies did not report significant improvement in HRQOL in pregabalin treatment groups as measured by the SF-36, although a numerical improvement was observed [17]. Other studies found significantly improved results on the SF-36 in higher doses (+300 mg/day) of pregabalin treatment [19,22]. In patients with PHN, significant improvement was observed in the physical component of the SF-36 as compared to the mental health component [25]. Conversely, other studies reported significant improvements in all components of SF-36 for both 150 and 300 mg/day pregabalin treatment groups [26]. Another study by Vranken et al. [30] in patients with unspecified etiology of NeP showed significant improvement in only the mental health domain of the SF-36 survey and not the social or emotional domain.

In DPN patients, the Sheehan disability scale (SDS) was utilized to assess functional impairment across three interrelated domains: work/school, social, and family life, with lower scores meaning HRQOL [35]. Jiang et al. [18] reported significant reductions in SDS scores, suggesting improved HRQOL for the pregabalin treatment group compared to placebo. However, these improvements were not significantly different when compared to other NeP treatments such as duloxetine and gabapentin [24]. Other QOL measures in DPN patients included the quality-of-life questionnaire-diabetic neuropathy version (QOL-DN), designed to capture the physiological symptoms of DPN along with influences on daily living with negative values indicating improvement [36]. A single study using the QOL-DN found significant improvements in certain domains for the pregabalin treatment group, although not across all domains [20]. Another frequently used tool for clinical trials to measure HRQOL included the EuroQol five-dimension (EQ-5D) [37], which measures multiple attributes including mobility, self-care, usual activities, pain/discomfort, and anxiety/depression. In DPN patients, significant improvements in EQ-5D scores were observed only in the higher dosage pregabalin group (600 mg/day) compared to placebo [25]. Similar findings were reported in other populations with NeP [30]. Finally, a single study by Huffman et al. [25] utilized the benefit, satisfaction, and willingness to continue measure, which comprises three single-item measures to gauge patients’ perceptions of treatment benefit, satisfaction, and willingness to continue therapy. This study found no significant differences in positive responses towards pregabalin compared to placebo.

Risk of Bias

**Figure 2:**
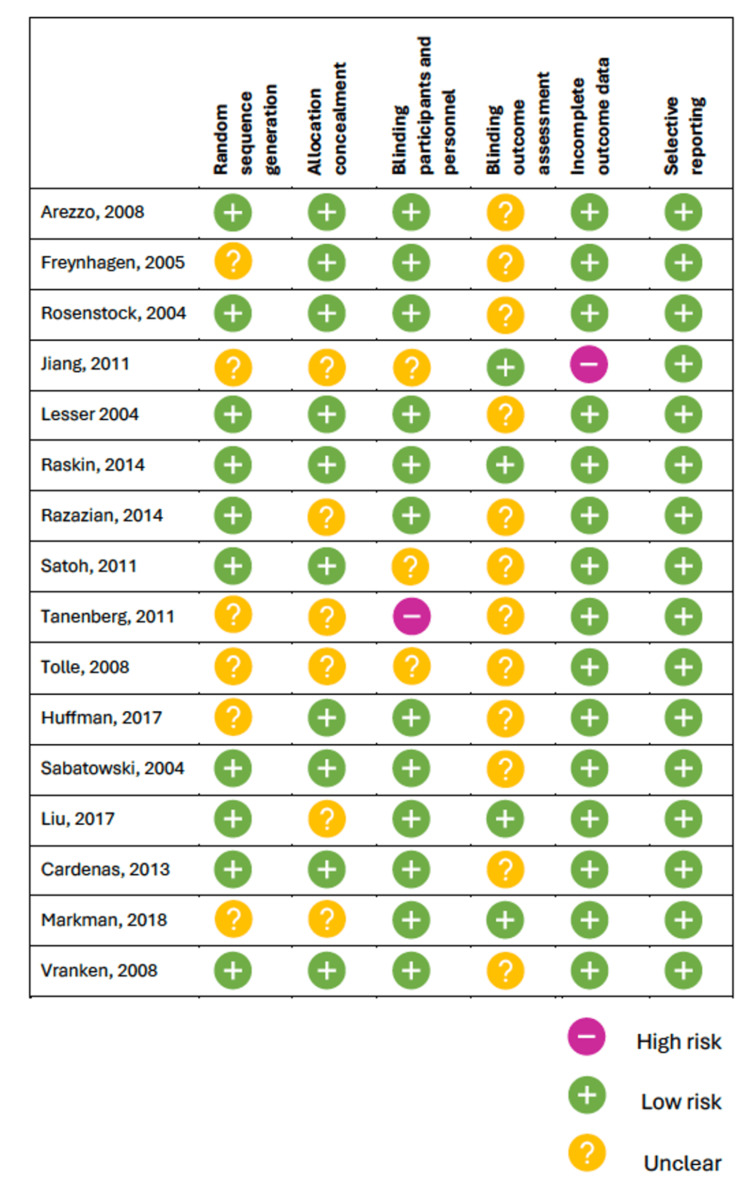
Risk of bias assessment for RCTs included All included studies [15-30] assessed the risk of bias, as shown above. RCTs: randomized controlled trials

The results of the risk of bias assessment for the included randomized, placebo-controlled RCTs are displayed in Figure 2. The results show that overall, the studies included were at low risk of bias. Most studies, however, were unclear in the blinding of their outcome assessment. This may be primarily due to underreporting of trial methodology associated with outcome assessment, making it difficult for the reader to assess it critically. Other sources of bias could be stemming from a lack of reporting on the process of random sequence generation for treatment groups [16,18,23-25,29]. Tanenberg et al. [23] did not blind participants and personnel to treatment being given and, therefore, is susceptible to bias in the highly subjective outcomes being discussed, including pain and other PROs. Further, one study [18] was found to be at risk for incomplete outcome data due to a high drop-out rate, which may have reduced the power of analysis. Study authors reported that the high drop-out within the trial may have been due to the patient's inability to adhere to intense and frequent assessments during the study period.

Discussion

Here, we have systematically reviewed the literature examining the effects of pregabalin on NeP and its impact on PROs. The phase III randomized trials included in this review assess various PROs that have been used when treating NeP with well-tolerated pregabalin. These include PROs of sleep interference, mood, anxiety, and depression, and HRQOL measures. The results highlight the significant role of PROs in understanding patient conditions and the complex relationship between pain and other outcome measures. Although relationships between more complicated measures of HRQOL remain to be further explored, to our knowledge, this review represents the first comprehensive synthesis of pregabalin's effects on PROs beyond pain.

Overall, results demonstrated a strong relationship between treatment with pregabalin and reduction in sleep interference related to NeP, as reported by study participants. Sleep disturbances are significant comorbidities in NeP patients and can feed into each other’s intensity [38]. Therefore, the observed improvement in sleep with pregabalin treatment is a crucial clinical outcome, especially given that benefits can be seen as early as one week into the trial period [15]. This is supported by previous studies that show increased non-rapid eye movement sleep in rats when treated with pregabalin [39]. This is likely due to the mechanism in the pharmacological action of pregabalin, which involves binding to voltage-gated calcium channels in the central nervous system, resulting in reduced excitatory neurotransmitters. This mechanism contributes to the analgesic effects and helps consolidate sleep, thereby reducing sleep disturbances [40]. Literature has supported pregabalin's effect on sleep improvement for other neurological conditions like epilepsy and fibromyalgia [41, 42]. Furthermore, the results indicate a dose-response relationship, with higher doses (+300 mg/day) associated with better outcomes in sleep interference. Improvements in sleep, as measured by patient-reported sleep diaries using the NRS, were observed regardless of the etiology of NeP.

A recent literature review has established that the 11-point NRS scale is an optimal and valid tool for measuring pain and sleep interference in adult patients [43]. Although a majority of studies used this self-reported measure to assess sleep interference, other studies also utilized standardized tools to measure sleep disturbance. Among these, the MOS-SS is notable for its strong psychometric properties and sensitivity to changes in patients with NeP of various etiologies, as demonstrated in the studies reviewed in this report [44]. Sleep quality measured via the MOS-SS showed improved scores in pregabalin treatment groups as compared to placebo and were statistically significant for all NeP etiological groups of studies except for PHN. The lack of significant results in PHN patients shows that a complex relationship may exist between pain and comorbid sleep disturbance as the psychosocial aspects of NeP etiologies may differ. Treatment strategies may need to consider not only the primary condition (i.e., NeP) but also comorbid conditions to restore overall quality of life [45].

Overwhelming evidence was not found for improvements in measurements of depression, where the influences of pregabalin treatment varied between studies, types of tools used to measure depression, and even dosage of pregabalin treatment. However, it should be noted that although many measures for depression did not yield significant results in pregabalin treatment groups as compared to placebo, all measures did record a numerical improvement in scores measuring depressive symptoms in pregabalin treatment groups. This shows the deleterious effects that conditions such as DPN and PHN can have on mood and mental health [26]. Information from more primary studies focusing on the relationship between NeP, pregabalin treatment, and depressive symptoms is required.

Anxiety disorders are prevalent in the general population, and the likelihood of developing the anxiety disorder increases four times in NeP [46]. In the current reviewed studies, significant improvements in anxiety symptoms in pregabalin treatment groups as compared to placebo were observed. This is likely due to the anxiolytic effects of pregabalin as seen in other controlled studies of patients with anxiety disorders [47]. This highlights the clinical importance of pregabalin in treating anxiety, independent of pain relief. The findings of this review emphasize the importance of the etiology of NeP when evaluating the improvement in anxiety symptoms from pregabalin treatment. Understanding the psychosocial aspects of the disease, such as NeP resulting from traumatic events as compared to DPN, could prove to be clinically important when considering treatment options. Indeed, psychosocial aspects of NeP can influence the severity, coping, adaptation, and stress response to the pain being experienced [48]. Moreover, simply having a chronic condition can influence stress and produce anxiety symptoms, making it difficult to diagnose and measure anxiety within a population experiencing pain [49]. Overall, psychiatric comorbidities in a NeP patient population can confuse the understanding of cause and effect between pain and anxiety/depression, but from a clinical standpoint, this may be irrelevant as the ultimate impact of psychiatric issues on NeP progression and patient response to treatment is important when creating effective multimodal treatment plans.

Overall, the results of the review show mixed evidence in terms of improvements in HRQOL measures. Studies assessing DPN patients do show improvement in certain HRQOL measures and show this improvement for higher doses of pregabalin versus lower. Further, when comparing pregabalin treatment with other NeP treatments, no significant difference was seen in HRQOL measures. Although data is limited, NeP is known to decrease the quality of life, including social functioning [10]. Studies reviewed in this report utilize a variety of validated tools and questionnaires to measure QOL before and after pregabalin treatment. The lack of statistically significant evidence in treated patients in improvements in HRQOL may be due to patients experiencing reduced QOL before entering the study and were not able to report sufficient improvement in QOL measures during the study period. Indeed, the mean duration of NeP in patients who seek treatment is seven years, which is a lengthy time for chronic pain to negatively influence the different aspects of their lives [50]. In the studies reviewed in this report, the mean age of study participants ranged from 55 to 73, which means that participants likely joined the study with a long history of experiencing pain and its influences on their daily functioning, social context, and adaptation to disease. Studies, especially of the duration reviewed here, may not be sufficient to capture and adequately measure HRQOL in NeP patient populations, where more data collection on patient histories and the long-term influence of NeP may be needed to assess treatment options.

The results of this review should be seen in light of certain limitations. Due to the heterogeneity in outcome measurements and the use of augmented tools to measure certain PROs, a meta-analysis of the results was difficult to do; therefore, the results only present a narrative analysis. Additionally, the search strategy for this review, focusing on four primary databases, may not have been wide-reaching enough to capture all primary literature, specifically the lack of epidemiological studies, on the topic of interest. Furthermore, the decision to exclude cross-over RCTs may have limited the amount of evidence available for review. A common limitation across the studies reviewed was study duration, where participants in the trials may not have received pregabalin for a sufficient amount of time to see treatment effects on PROs. Expanding the inclusion criteria or conducting further reviews focusing on longitudinal epidemiological studies may be needed to effectively evaluate the treatment effects of pregabalin. Finally, with the focus of this review on an adult population, generalizability may be limited. Despite these limitations, this review finds its strengths in highlighting and summarizing the important PROs in the treatment of NeP with pregabalin from a diverse patient population.

## Conclusions

This review of RCTs assessing the impact of pregabalin on PROs in NeP offers a comprehensive overview of various PRO measurements across a diverse population of NeP patients. The findings highlight the complex, multidimensional effects of NeP on patients' sleep, psychological health, and overall quality of life. The review underscores the challenges of measuring these outcomes in individuals with chronic pain and suggests that adopting a more holistic approach to understanding patient experiences could enhance insights into how different aspects of life are affected by pain. Future reviews and studies should focus on exploring the long-term experiences of NeP patients through extended, multidimensional study designs. Such research could contribute to developing multimodal treatment strategies that more effectively improve the quality of life for NeP patients.

## Appendices

**Table 4:** Search strategy

Step	Strategy
1	Peripheral neuropathy
2	Central neuropathy
3	Neuropathic pain
4	Neuralgia
5	Painful neuralgia
6	Painful neuropathy
7	Postherpetic neuralgia
8	Diabetic neuropathy
9	#1 OR 2 OR 3 OR 4 OR 5 OR 6 OR 7 OR 8
10	Pregabalin
11	Lyrica
12	3-Isobutyl GABA
13	3-Isobutyl GABA
14	#10 OR 11 OR 12 OR 13
15	Patient-reported outcome
16	Quality of life
17	QOL
18	Pain score
19	Health-related quality of life
20	HRQL
21	Mood
22	Psychological health
23	Physical health
24	Mental health
25	Health status
26	PRO
27	#15 OR 16 OR 17 OR 18 OR 19 OR 20 OR 21 OR 22 OR 23 OR 24 OR 25 OR 26
28	#9 AND 14 AND 27
Filters	Human, article, English article type: clinical trial, phase III clinical trial, protocol, comparative study, controlled clinical trial, randomized controlled trial, clinical conference, clinical study, clinical trial, preprint, publication dates: 01/01/2000-08/14/2024
